# Systematic Literature Review of Role of Noroviruses in Sporadic Gastroenteritis

**DOI:** 10.3201/eid1408.071114

**Published:** 2008-08

**Authors:** Manish M. Patel, Marc-Alain Widdowson, Roger I. Glass, Kenichiro Akazawa, Jan Vinjé, Umesh D. Parashar

**Affiliations:** * Centers for Disease Control and Prevention, Atlanta, Georgia, USA; † National Institutes of Health, Bethesda, Maryland, USA; ‡ Chigasaki Tokushukai Medical Center, Kanagawa, Japan

**Keywords:** norovirus, Norwalk virus, calicivirus infections, burden of illness, diarrhea, gastroenteritis, epidemiology, research

## Abstract

We conducted a systematic review of studies that used reverse transcription–PCR to diagnose norovirus (NoV) infections in patients with mild or moderate (outpatient) and severe (hospitalized) diarrhea. NoVs accounted for 12% (95% confidence interval [CI] 10%–15%) of severe gastroenteritis cases among children <5 years of age and 12% (95% CI 9%–15%) of mild and moderate diarrhea cases among persons of all ages. Of 19 studies among children <5 years of age, 7 were in developing countries where pooled prevalence of severe NoV disease (12%) was comparable to that for industrialized countries (12%). We estimate that each year NoVs cause 64,000 episodes of diarrhea requiring hospitalization and 900,000 clinic visits among children in industrialized countries, and up to 200,000 deaths of children <5 years of age in developing countries. Future efforts should focus on developing targeted strategies, possibly even vaccines, for preventing NoV disease and better documenting their impact among children living in developing countries, where >95% of the deaths from diarrhea occur.

## Abstract 2

Noroviruses accounted for 12% of severe gastroenteritis cases among children <5 years of age.

Despite improved safety of food, water, and sanitation and aggressive promotion of noninvasive interventions (e.g., oral rehydration therapy) and prevention strategies (e.g., increased breastfeeding), diarrhea remains a common cause of illness worldwide. It accounts for ≈1.8 million annual deaths in children <5 years of age (*1*). Reduction of this disease will require targeted prevention and treatment strategies against the common agents causing severe diarrhea.

Noroviruses (NoVs) and sapoviruses are genetically and antigenically diverse single-stranded RNA viruses that belong to 2 different genera (*Norovirus* and *Sapovirus)* in the family *Caliciviridae* and are collectively referred to as human caliciviruses (*2*). The prototype virus of the NoVs, Norwalk virus, was identified in 1972. However, the inability to cultivate these viruses in routine cell culture and the consequent challenges in developing sensitive nonmolecular diagnostic assays hindered initial efforts to define the epidemiology and assess the impact of disease associated with NoV infection. In the past 15 years, the availability of sensitive molecular diagnostic methods based on reverse transcription–PCR (RT-PCR) has allowed broader examination of the etiologic role of NoVs in epidemic and sporadic gastroenteritis (*3*,*4*).

Since the application of molecular assays, NoVs have been well-documented as the leading cause of epidemic gastroenteritis in all age groups, causing >90% of nonbacterial and ≈50% of all-cause epidemic gastroenteritis worldwide (*5*). Recent studies that used improved diagnostics have demonstrated that NoVs may also fill in the “diagnostic gap” in severe sporadic gastroenteritis among all age groups worldwide (*6*). Much of the misconception of NoV as an infrequent cause of severe sporadic diarrhea might also stem from studies undertaken before the mid-1990s that found low rates of NoV infection because available diagnostics such as electron microscopy and antigen detection assays had poor sensitivity. Recent data are emerging that are debunking these misconceptions, suggesting that the impact of NoV disease may be much greater than previously suspected and the disease may be more severe in some populations (*4*,*6*–*8*). However, because these novel assays are not typically available outside of reference laboratories, the true global prevalence and potential economic impact of NoV disease remain unrecognized (*3*). To further understand the etiologic role of NoVs in sporadic diarrhea, we conducted a systematic review to identify studies that used similar inclusion criteria and molecular assays based on RT-PCR to detect NoVs in fecal specimens from patients with diarrhea.

## Methods

We searched MEDLINE, EMBASE, and Google Scholar to identify studies published in English between January 1990 and February 2008. We used the following keywords: *Norwalk*, *norovirus,* *Norwalk-like virus,* *human calicivirus,* *calicivirus,* *NLV,* *small round virus,* and *small round structured virus*. We reviewed all abstracts to identify articles that assessed the prevalence of NoV among sporadic cases of diarrhea. To ensure complete capture of all relevant studies, we cross-referenced all articles from the bibliography of the selected articles. After reviewing each article, we selected studies that met the following inclusion criteria: 1) study duration was >1 year, and 2) study used RT-PCR to diagnose caliciviruses (NoV and sapovirus) or NoV in patients with diarrhea. We included studies that tested for caliciviruses, even when they did not differentiate between NoV and sapovirus. For these studies, we multiplied the proportion of caliciviruses detected in each study by the mean proportion of caliciviruses that were NoV among studies that differentiated between NoVs and sapoviruses to yield the estimated NoV prevalence in each study. We excluded studies that did not provide a denominator (i.e., the total number of patients with diarrhea in the study population) or that only conducted molecular analysis using a fraction of the fecal samples (Technical Appendix 1). If the authors presented the data again in another study, only 1 study was included. See Technical Appendix 2, for a list of all references used in the review but not cited in this article.

We stratified studies into 2 settings: community or clinic-based (mild or moderate diarrhea) and hospital-based including emergency department and inpatients (severe diarrhea). We counted cases in which NoV was detected in the presence of >1 other pathogens (i.e., mixed infection) as NoV infection; however, we also present data on mixed infections, when available. Pooled proportions and 95% confidence intervals (CIs) of NoV-positive cases were calculated by using the random effects models (DerSimonnian and Laird method, StatsDirect Ltd, Cheshire, UK). For the studies that included fecal testing on concurrent diarrhea-free controls, the pooled proportions were based on absolute difference in NoV detection rate between cases and controls, thus only including the fraction of cases attributable to NoVs. The Cochran Q statistic and degrees of freedom (df) are presented as a measure of heterogeneity among studies. Analyses were conducted with StatsDirect version 2.5.7 (StatsDirect Ltd).

To calculate the number of outpatient NoV episodes and hospitalizations for children living in industrialized countries (where 23 of 31 studies in our review were conducted), we multiplied the total number of estimated diarrhea episodes in each clinical setting by the pooled proportion attributable to NoV based on the studies we reviewed to yield the number of NoV cases in each setting (*9*). No data exist on estimates of total diarrhea episodes in industrialized countries. Thus, we divided the estimates of outpatient and inpatient rotavirus episodes for industrialized countries, provided by Parashar et al. (*9*), by the proportion of diarrhea episodes attributable to rotavirus (23% and 42%, respectively) in the United States (*10*) and Europe (*11*) to yield the annual number of total diarrhea episodes in industrialized countries. To estimate the proportion of outpatient (23%) and inpatient (42%) diarrhea episodes attributable to rotavirus, we assumed the midpoint of the proportion of gastroenteritis visits attributable to rotavirus in the United States (19% and 35%, respectively) and 7 European countries (27% and 50% respectively) for each setting, respectively.

To estimate NoV-associated deaths and hospitalizations among children in developing countries, we multiplied global estimates of diarrhea deaths (*1*) and hospitalizations (*9*) by the pooled proportion of NoV among children <5 years of age hospitalized with diarrhea. Data from developing countries were sparse on fraction of NoV-associated diarrhea episodes in the outpatient setting.

## Results

Overall, we reviewed 235 studies and identified 31 original studies that met our inclusion criteria (Tables 1, 2) (*6*,*12*–*41*). Of these 31 studies, 20 were conducted in high-income countries, 2 were high-middle-income countries, 5 were low-middle income, and 4 were low-income countries, based on World Bank classification of economies. The duration of these studies was 1–5 years. Fourteen studies tested only for NoV (*13*,*14*,*19*,*21*,*22*,*27*,*29*,*30*,*35*,*37*–*41*); 17 tested for NoV and sapovirus (*6*,*12*,*15*–*18*,*20*,*23*–*26*,*28*,*31*–*34*,*36*). Among 13 of 17 studies that tested for and presented separate detection rates on both caliciviruses, NoV was detected in 84.5% and 88.5% of the community- and hospital-based studies, respectively (*6*,*15*,*16*,*20*,*23*–*26*,*31*–*34*,*36*). In these 13 studies, 69%–90% of the outpatient cases and 61%–100% of the hospital cases with caliciviruses were identified as NoV.

**Table 1:** Summary of studies examining prevalence of NoV in persons with severe sporadic AGE, using RT-PCR for studies >12 months’ duration, community and outpatient clinics*

Ref	Country	Study duration, mos	Age group, y	No. AGE cases	No. NoV positive (single and mixed)	% NoV positive	No. mixed pathogens	% Mixed pathogens	No. control patients	% Control patients positive for NoV
(*6*)†	England	12†	All	2,422	871	36.0	–‡	–	2,205	16.2
(*12*)§	France	26	<13	414	49	11.8	27	6.5	50	0.0
(*15*)	Netherlands	12	All	709	114	16.1	–	–	669	5.2
(*16*)	Netherlands	36	All	857	43	5.0	–	–	574	1.0
(*13*)*¶*	Hong Kong	12	All	995	92	9.2	–	–	–	–
(*14*)	Australia	17	All	638	73	11.4	7	1.1	–	–
(*33*)*¶*	India	36	<5	500	38	7.6	5	1.0	173	4.0
(*18*)§*¶*	Chile	31	<5	274	15	5.5	–	–	–	–
(*17*)§	Finland	21	<2	1,477	264	17.8	13	0.9	47	0.0
(*20*)	Japan	12	<11	557	106	19.0	12	2.2	–	–
(*21*)	Japan	12	<11	402	58	14.4	–	–	–	–
(*19*)	Japan	12	<5	752	139	18.5	3	0.4	–	–
(*34*)¶#	Tunisia	15	<12	380	49	12.9	13	3.4	–	–

*NoV, norovirus; AGE, acute gastroenteritis; RT-PCR, reverse transcription–PCR; Ref, reference; –. not assessed in the study. Studies were of >12 months’ duration. †Using RT-PCR, tested stored specimens from a previous study that used electron microscopy for NoV detection; enrollment was staggered over 29 months with 70 clinics, each enrolling patients for 12 months. ‡Tested for mixed pathogens but did not provide pathogen-specific results. §NoV prevalance in these studies was estimated by multiplying the calicivirus prevalance by average proportion of caliciviruses determined to be NoV (84.5%) among outpatient studies reporting NoV and sapovirus results separately. ¶O’Ryan et al. (*18*) included clinic-, emergency department–, and hospital-based patients; Lau et al. (*13*), Monica et al. (*33*), and Sdiri-Loulizi et al. (*34*) included community/clinic- and hospital-based patients. Only community and clinic data are presented here. #Age- and setting-specific data were obtained through personal communication with the author.

**Table 2:** Summary of studies that examined prevalence of NoV in persons with severe sporadic AGE, emergency department visits and hospitalizations, by using RT-PCR for >12 months*

Ref	Country	Study duration, mo	Age group, y	No. AGE cases	No. NoV positive (single and mixed)	% NoV positive	No. NoV positive (mixed)	% Mixed	No. control patients	% Controls positive for NoV
Emergency department										
(*22*)	Spain	12	<14	363	16	4.4	1	0.3	–	–
(*18*)†‡	Chile	25 and 17§	<5	248	23	9.3	–	–	80	1.3
Hospital										
(*35*)	Italy	12	<3	365	93	25.5	35	9.6	–	–
(*23*)	Malawi	12	<5	398	26	6.5	12	3.0	–	–
(*24*)	Vietnam	12	<15	1,339	72	5.4	0	0.0	–	–
(*25*)	Thailand	12	<5	105	8	7.6	–	–	–	–
(*36*)	Thailand	20	<5	248	35	14.1	–	–	–	–
(*26*)	Australia	60	<5	1,233	108	8.8	–	–	–	–
(*13*)‡	Hong Kong	12	All	735	123	16.7	–	–	–	–
(*37*)	South Korea	24	<5	962	132	13.7	18	1.9	–	–
(*33*)‡	India	36	<5	350	53	15.1	26	7.4	173	4.0
(*27*)	Germany	12	<16¶	217	45	20.7	–	–	50	4.0
(*38*)	Japan	24	Children	500	66	13.2	2	0.4	–	–
(*18*)‡	Chile	25 and 17§	<5	162	8	4.9	–	–	50	0.0
(*39*)	Madagascar	13	<5	237	14	5.9	0	0.0	–	–
(*27*)†	Peru	24	<5	233	72	30.7	56	23.9	248	11.4
(*29*)	Spain	12	<5	656	79	12.0	36	5.5	–	–
(*30*)	Australia	36	<5	360	9	2.5	–	–	–	–
(*34*)‡#	Tunisia	27	<12	252	61	24.2	12	4.8	–	–
(*40*)	Brazil	12	<5	318	65	20.4	14	4.4	–	–
(*31*)	South Africa	48	All	1,296	32	2.5	–	–	–	–
(*41*)	South Korea	12	<5	762	114	15.0	–	–	–	–
(*31*)	USA	24	<4	1,840	131	7.1	–	–		–

*NoV, norovirus; AGE, acute gastroenteritis; RT-PCR, reverse transcription–PCR; Ref, reference; –, not assessed in the study. †NoV prevalence (total and mixed alone) in these studies was estimated by multiplying the calicivirus prevalence by average proportion fo caliciviruses determined to be NoV (88.5%) among hospital-based studies reporting NoV and sapovirus results separately. ‡O’Ryan et al. (*18*) included clinic-, emergency department–, and hospital-based patients; Lau et al. (*13*), Monica et al. (*33*), and Sdiri-Loulizi et al. (*34*) included community/clinic- and hospital-based patients. Only emergency department and hospitalization data are presented here. §Included 2 hospitals with 25- and 17-month enrollment periods. ¶98% (213 of 217) of the case-patients were <5 y of age. #Age- and setting-specific data were obtained through personal communication with the author.

### Illness by Age and Setting

#### Community- or Clinic-based Cases (i.e., Mild and Moderate Diarrhea)

**Figure 1:**
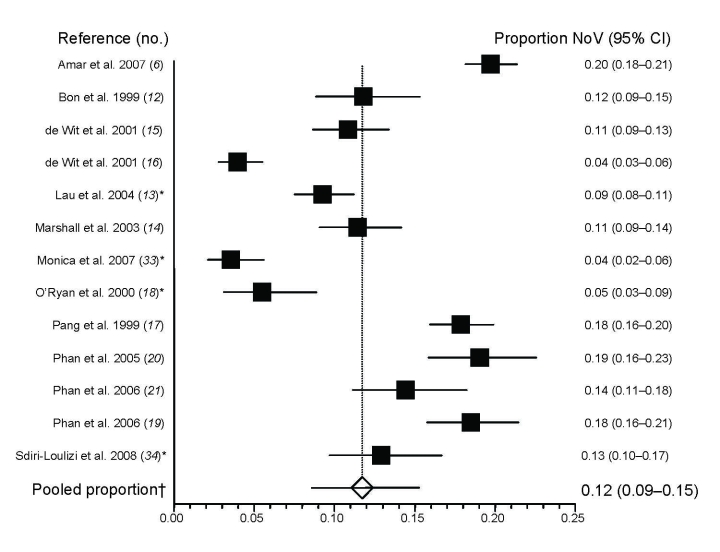
Summary of studies assessing proportion of norovirus (NoV)-positive fecal samples among persons with community and outpatient cases of sporadic diarrhea (all ages). *Lau et al. (*13*), O’Ryan et al. (*18*), Monica et al. (*33*), and Sdiri-Loulizi et al. (*34*) included outpatient and emergency department/hospital patients, but only outpatient data are included in this figure. †Pooled proportion calculated by using the random effects model (DerSimonian and Laird method, StatsDirect Ltd, Cheshire, UK). For studies that included controls, prevalence of NoV among controls was subtracted from prevalence of NoV among case-patients. CI, confidence interval.

Among the 13 studies of community- or clinic-based diarrhea cases, NoVs were detected in 5%–36% of cases; pooled proportion was 12% (95% CI 9%–15%; Cochran Q 335; df 12) (Figure 1). Five studies enrolled both adults and children, and 8 studies focused only on children, each with varying age ranges (overall range 0–13 years). In the 8 studies that assessed mixed infections, 0.4%–6.5% (median 1.1%) of the gastroenteritis cases were also positive for another viral or bacterial pathogen (Table 1).

#### Hospitalizations (i.e., Severe Diarrhea)

**Figure 2:**
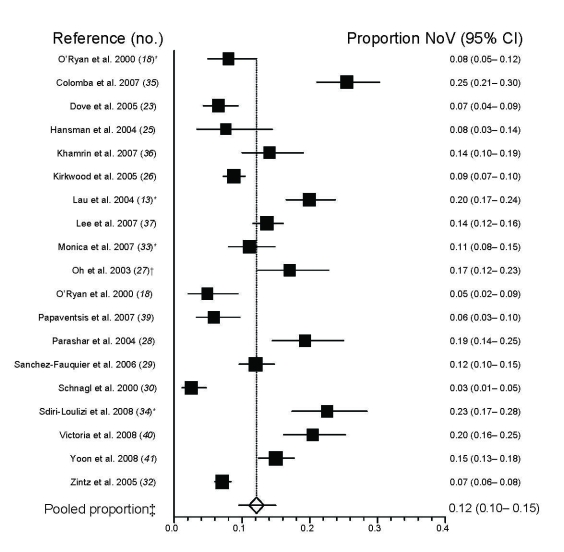
Summary of studies assessing proportion of norovirus (NoV)-positive fecal samples among hospitalized and emergency department cases of children <5 years of age who had sporadic diarrhea. *Lau et al. (*13*), O’Ryan et al. (*18*), Monica et al. (*33*), and Sdiri-Loulizi et al. (*34*) included outpatient and emergency department/hospital patients, but only inpatient data are included in this figure. †Oh et al. (*27*), 98% (213 of 217) of the case-patients were <5 years of age. ‡Pooled proportion calculated using the random effects model (DerSimonian and Laird method (StatsDirect Ltd, Cheshire, UK). For studies that included controls, prevalence of NoV among controls was subtracted from prevalence of NoV among case-patients. CI, confidence interval.

Twenty-three studies [hospital-based (n = 21); emergency department–based (n = 1); both (n = 1)] evaluated NoV disease among hospitalized diarrhea case-patients in whom the proportion of NoV disease ranged from 3% to 31%; pooled proportion was 11% (95% CI 8%–14%). Most (n = 19) of these studies of severe diarrhea cases focused on children <5 years of age, and the pooled proportion of NoV disease in these studies was 12% (95% CI 10%–15%) (Figure 2). In the 5 studies that assessed for multiple enteric pathogens, mixed infections were detected in 0%–24% (median 3.7%) of the gastroenteritis cases (Table 2).

Most (>95%) of the world’s diarrheal deaths occur in low-middle– and low-income countries. In our review, 7 of 19 studies assessing prevalence of severe NoV disease among children <5 years of age were conducted in these countries (*23*,*25*,*28*,*33*,*34*,*36*,*39*). Pooled proportions for NoV-associated childhood hospitalization were 12% (95%CI 8%–17%) among low-middle and low income in comparison to 12% (95% CI 9%–16%) for high- and high-middle–income countries.

### Studies with Concurrent Controls

Concurrent diarrhea-free controls were enrolled in 9 of 31 studies, and NoVs were detected in 0%–16% (median 4%) of the controls (*6*,*12*,*15*–*18*,*27*,*28*,*33*). On the basis of the difference in detection rate between cases and controls in these studies, we estimate that the fraction of cases attributable to NoV in these studies was 4%–20% (median 12%) for mild to moderate diarrhea and 2%–26% (median 11%) for severe diarrhea.

### Strain Characterization

Overall, 19 studies characterized the NoV strains by using nucleotide sequencing (*13*,*14*,*17*,*19*–*21*,*23*,*24*,*26*,*27*,*32*–*36*,*38*–*41*). Among NoV cases, strains belonging to NoV genogroup (G) II were the most common (range 75%–100%). With the exception of 2 studies, the overwhelming majority of the NoV strains belonged to the GII.4 cluster (*21*,*23*). In Malawi (1 of the 4 low-income countries in our analysis), GII.3 strains were detected in 69% of the samples that were sequenced (*23*). Similarly, in 2006, novel GII.3 strains were identified in 44% of the samples in a clinic-based study in Japan (*21*). Through genetic characterization, the authors demonstrated that these GII.3 strains likely were recombinant strains that appeared over a period of 4 months.

### Estimated Prevalence of NoV Disease in Children

We estimate that each year NoVs cause ≈900,000 episodes of gastroenteritis that require a clinic visit and ≈64,000 hospitalizations among children <5 years of age residing in high-income countries (Table 3). If one assumes that the proportion of annual childhood diarrhea-associated hospitalizations (≈124 million) and deaths (≈1.8 million) in developing countries approximates the overall proportion of children (12.1%) with severe NoV illness in our review, NoVs may cause up to 1.1 million hospitalizations and 218,000 deaths each year in children in developing countries.

**Table 3:** Estimates of annual number of episodes of norovirus-associated diarrhea among children <5 years of age in industrialized and developing countries, by setting

Setting	Annual no. diarrhea-associated events*	Pooled proportion of episodes attributable to noroviruses, %	Total no. norovirus episodes	Annual incidence per 100,000 children†‡
Industrialized countries†				
Outpatient	7,743,000	11.7	906,000	1,665
Inpatient	531,000	12.1	64,200	118
Developing countries‡§				
Inpatient	9,015,000	12.1	1,091,000	197
Deaths	1,800,000	12.1	218,000	39

*Refer to Methods section of article. †Industrialized countries’ incidence based on UNICEF 2003 population estimates of 54,425,000 children <5 y of age in developing countries (www.unicef.org/sowc05/english/Developingregional.html). ‡Developing countries’ incidence based on UNICEF 2003 population estimates of 552,742,000 children <5 y of age in industrialized (www.unicef.org/sowc05/english/Industrializedregional.html) §Data from developing countries were sparse on fraction of norovirus-associated diarrhea episodes in the outpatient setting.

## Discussion

This systematic review of studies that used RT-PCR for detection of NoVs in fecal specimens clearly indicates that these viruses play an important role in the cause of both mild and severe gastroenteritis worldwide. In 1979, Greenberg et al. demonstrated that virtually all children in various countries worldwide acquired antibodies to NoVs by 5–15 years of age (Technical Appendix 2). However, despite strong evidence that NoV infection was ubiquitous, detection rates in children hospitalized with diarrhea were low in early studies that used electron microscopy and antigen assays (*4*). The advent of conventional RT-PCR for the diagnosis of NoVs has substantially changed our understanding of their epidemiology. Among all reported studies that used conventional RT-PCR, NoVs were detected in ≈12% of children <5 years of age with severe diarrhea, which suggests that these viruses are the second most common cause of severe childhood gastroenteritis, following rotavirus. In addition, although some studies suggest that NoV infections in the community are slightly less severe than rotavirus infections, data also exist to suggest that these childhood infections may be similar in severity, which may particularly apply to hospitalized children (Technical Appendix 2). On the basis of the pooled detection rates of NoV in our review, we would estimate that in the United States alone NoVs may account for >235,000 clinic visits, 91,000 emergency room visits, and 23,000 hospitalizations among children <5 years of age (*10*). Limited data from developing countries are available to make firm estimates, but NoV disease may cause >1 million hospitalizations and 200,000 deaths each year among children <5 years of age.

Although these figures provide a preliminary indication of the substantial magnitude of illness from NoV disease, they may underestimate the true extent of disease. Evidence suggests that detection of NoVs in fecal specimens by conventional RT-PCRs may be limited by factors such as low virus concentrations in feces, improper specimen storage, inefficient viral RNA extraction, presence of fecal reverse transcriptase inhibitors, and use of different primers (Technical Appendix 2). In addition, NoVs are extremely genetically diverse and none of the reported conventional RT-PCR assays is able to detect all strains (Technical Appendix 2). These hypotheses are supported by the findings of an evaluation of children with gastroenteritis in Peru in which both RT-PCR testing of fecal specimens and serologic assays were used to assess NoV infection, and serologic testing was found to increase the rates of NoV detection from 35% with fecal testing alone to 55% by use of either assay (*28*). A recent validation study comparing state-of-the-art real-time RT-PCR with conventional RT-PCR found that the sensitivity of real-time RT-PCR was greater than that of the conventional method, especially for samples containing low NoV concentrations or RT-PCR inhibitors (Technical Appendix 2). The broader application of real-time RT-PCR assays to diagnose NoV among children hospitalized with gastroenteritis should provide better estimates of the true prevalence of disease.

Some factors could have led us to overestimate the extent of sporadic NoV disease. Our estimates of sporadic NoV disease prevalence are based on a review of active surveillance data of all diarrhea cases and do not exclude cases originating from an outbreak. In a few studies, NoVs were detected in patients who were co-infected with another pathogen, and only a limited number of studies enrolled concurrent healthy controls, thus making it difficult to determine the fraction of diarrhea cases truly attributable to NoV. Among all studies testing for co-infections, however, the median rate of detecting another pathogen in addition to NoV was low (2%). This finding, combined with the fact that most studies only assessed for NoV among samples that previously tested negative for other bacterial and viral pathogens, suggests that our overall pooled proportion attributable to NoVs is unlikely to be much lower. In addition, for the studies that enrolled diarrhea-free controls, we subtracted control prevalence from the case prevalence of NoV disease when calculating the overall pooled estimate. Lastly, other unmeasured factors underestimating disease prevalence that could not be accounted for, such as inefficient primers, low virus shedding, delays in specimen collection, and lack of a sensitive case-definition are also likely to exist in these studies.

The heterogeneity in the NoV literature is evident and should be considered when interpreting the results of this review. Our systematic approach and strict inclusion criteria likely reduced heterogeneity but do not eliminate biases in the original studies, diversity in study design and population, and publication bias. Nonetheless, the findings of this review suggest that NoVs are a frequent cause of mild and severe sporadic gastroenteritis among children in high- and middle-income countries. In addition, hospital studies in our review only assessed patients admitted with diarrhea. Because of the highly infectious nature of NoVs, substantial additional health and economic effects would also occur from nosocomial disease and outbreaks in healthcare facilities, as previously identified by Lopman et al (*7*). NoVs are also a frequent cause of severe illness and death from diarrhea among children in developing countries, although firm conclusions cannot be made because of limited data. Systematic evaluations that use broadly reactive, state-of-the-art diagnostic assays, with concurrent evaluation of healthy controls and examination of potential co-infection, are needed to fully understand the role of NoVs in the etiology of sporadic childhood gastroenteritis. These evaluations are especially necessary in developing countries, where diarrhea remains a leading cause of childhood death, causing >1.8 million annual deaths (*1*).

The increasing evidence documenting the magnitude of the NoV disease prevalence provides support for considering targeted interventions, such as vaccines, for reducing the extent of this illness among young children. However, if one considers that NoV frequently causes both sporadic and epidemic gastroenteritis and can affect all age groups, some other potential targets for vaccination may include elderly persons in nursing homes, who are vulnerable to severe complications, and military recruits, in whom sporadic and epidemic NoV disease is known to incur substantial illness and financial costs from work disruption (*7*; Technical Appendix 2). The development of vaccines against NoVs will likely be challenging because the immunity to these viruses and the diversity and evolution of circulating strains are incompletely understood (Technical Appendix 2). However, genotype II, cluster 4 NoV strains appeared to be by far the most prevalent strains among the studies we reviewed, and these strains may be the primary targets for vaccine development.

Carefully designed epidemiologic studies that evaluate NoV prevalence in children with diarrhea and a suitable comparison group and that use sensitive molecular assays will help further define target groups that would benefit from vaccines and other interventions. A particularly pressing need exists for better quantifying the extent of severe norovirus disease among children in developing countries and identifying prevention strategies to help reduce the prevalence of deaths from diarrhea in the poorest countries.

## Supplementary Material

Technical Appendix 1 References Excluded from Review

Technical Appendix 2 References Used in Review but Not Cited in Article
