# Supplementary material for: Systematic Literature Review of Role of Noroviruses in Sporadic Gastroenteritis
Source: Emerg Infect Dis. 2008 Aug;14(8):1224–31. doi: 10.3201/eid1408.071114 (PMC2600393; doi:10.3201/eid1408.071114)
Supplement: Technical Appendix 1 — References Excluded from Review [file 07-1114_Techapp1-s1.pdf]

Systematic Literature Review of Role of Human Noroviruses in Sporadic Gastroenteritis

Technical Appendix 1

References Excluded from Review

1. Al-Mashhadani MN, Nakagomi O, Dove W, Ahmed H, Nakagomi T, Hart CA, et al. Norovirus gastroenteritis among children in Iraqi Kurdistan. J Med Virol. 2008;80:506–9.

2. Armah GE, Gallimore CI, Binka FN, Asmah RH, Green J, Ugoji U, et al. Characterisation of norovirus strains in rural Ghanaian children with acute diarrhoea. J Med Virol. 2006;78:1480–5.

3. Bereciartu A, Bok K, Gomez J. Identification of viral agents causing gastroenteritis among children in Buenos Aires, Argentina. J Clin Virol. 2002;25:197–203.

4. Castilho JG, Munford V, Resque HR, Fagundes-Neto U, Vinje J, Racz ML. Genetic diversity of norovirus among children with gastroenteritis in Sao Paulo State, Brazil. J Clin Microbiol. 2006;44:3947–53.

5. Chen SY, Chang YC, Lee YS, Chao HC, Tsao KC, Lin TY, et al. Molecular epidemiology and clinical manifestations of viral gastroenteritis in hospitalized pediatric patients in Northern Taiwan. J Clin Microbiol. 2007;45:2054–7.

6. Fabiana A, Donia D, Gabrieli R, Petrinca AR, Cenko F, Bebeci D, et al. Influence of enteric viruses on gastroenteritis in Albania: epidemiological and molecular analysis. J Med Virol. 2007;79:1844–9.

7. Foley B, O'Mahony J, Morgan SM, Hill C, Morgan JG. Detection of sporadic cases of Norwalk-like virus (NLV) and astrovirus infection in a single Irish hospital from 1996 to 1998. J Clin Virol. 2000;17:109–17.

8. Fretz R, Herrmann L, Christen A, Svoboda P, Dubuis O, Viollier EH, et al. Frequency of Norovirus in stool samples from patients with gastrointestinal symptoms in Switzerland. Eur J Clin Microbiol Infect Dis. 2005;24:214–6.

9. Guntapong R, Hansman GS, Oka T, Ogawa S, Kageyama T, Pongsuwanna Y, et al. Norovirus and sapovirus infections in Thailand. Jpn J Infect Dis. 2004;57:276–8.

10. Hansman GS, Kuramitsu M, Yoshida H, Katayama K, Takeda N, Ushijima H, et al. Viral gastroenteritis in Mongolian infants. Emerg Infect Dis. 2005;11:180–2.

11. Iturriza Gomara M, Simpson R, Perault AM, Redpath C, Lorgelly P, Joshi D, et al. Structured surveillance of infantile gastroenteritis in East Anglia, UK: incidence of infection with common viral gastroenteric pathogens. Epidemiol Infect. 2008;136:23–33.

12. Kang G, Hale AD, Richards AF, Jesudason MV, Estes MK, Brown DW. Detection of 'Norwalk-like viruses' in Vellore, southern India. Trans R Soc Trop Med Hyg. 2000;94:681–3.

13. Kirkwood CD, Bishop RF. Molecular detection of human calicivirus in young children hospitalized with acute gastroenteritis in Melbourne, Australia, during 1999. J Clin Microbiol. 2001;39:2722–4.

14. Koopmans M, Vinj J, de Wit M, Leenen I, van der Poel W, van Duynhoven Y. Molecular epidemiology of human enteric caliciviruses in The Netherlands. J Infect Dis. 2000;181(Suppl 2):S262–9.

15. Levett PN, Gu M, Luan B, Fearon M, Stubberfield J, Jamieson F. Longitudinal study of molecular epidemiology of small round-structured viruses in a pediatric population. J Clin Microbiol. 1996;34:1497–501.

16. Liu C, Grillner L, Jonsson K, Linde A, Shen K, Lindell AT, et al. Identification of viral agents associated with diarrhea in young children during a winter season in Beijing, China. J Clin Virol. 2006;35:69–72.

17. Marie-Cardine A, Gourlain K, Mouterde O, Castignolles N, Hellot MF, Mallet E, et al. Epidemiology of acute viral gastroenteritis in children hospitalized in Rouen, France. Clin Infect Dis. 2002;34:1170–8.

18. Moyo SJ, Gro N, Kirsti V, Matee MI, Kitundu J, Maselle SY, et al. Prevalence of enteropathogenic viruses and molecular characterization of group A rotavirus among children with diarrhea in Dar es Salaam Tanzania. BMC Public Health. 2007;7:359.

19. Parks CG, Moe CL, Rhodes D, Lima A, Barrett L, Tseng F, et al. Genomic diversity of "Norwalk like viruses" (NLVs): pediatric infections in a Brazilian shantytown. J Med Virol. 1999;58:426–34.

20. Phan TG, Okame M, Nguyen TA, Maneekarn N, Nishio O, Okitsu S, et al. Human astrovirus, norovirus (GI, GII), and sapovirus infections in Pakistani children with diarrhea. J Med Virol. 2004;73:256–61.

21. Qiao H, Nilsson M, Abreu ER, Hedlund KO, Johansen K, Zaori G, et al. Viral diarrhea in children in Beijing, China. J Med Virol. 1999;57:390–6.

22. Ramirez S, De Grazia S, Giammanco GM, Milici M, Colomba C, Ruggeri FM, et al. Detection of the norovirus variants GGII.4 hunter and GGIIb/hilversum in Italian children with gastroenteritis. J Med Virol. 2006;78:1656–62.

23. Reither K, Ignatius R, Weitzel T, Seidu-Korkor A, Anyidoho L, Saad E, et al. Acute childhood diarrhoea in northern Ghana: epidemiological, clinical and microbiological characteristics. BMC Infect Dis. 2007;7:104.

24. Simpson R, Aliyu S, Iturriza-Gomara M, Desselberger U, Gray J. Infantile viral gastroenteritis: on the way to closing the diagnostic gap. J Med Virol. 2003;70:258–62.

25. Soares CC, Santos N, Beard RS, Albuquerque MC, Maranhao AG, Rocha LN, et al. Norovirus detection and genotyping for children with gastroenteritis, Brazil. Emerg Infect Dis. 2007;13:1244–6.

26. Subekti DS, Tjaniadi P, Lesmana M, Simanjuntak C, Komalarini S, Digdowirogo H, et al. Characterization of Norwalk-like virus associated with gastroenteritis in Indonesia. J Med Virol. 2002;67:253–8.

27. Traore O, Belliot G, Mollat C, Piloquet H, Chamoux C, Laveran H, et al. RT-PCR identification and typing of astroviruses and Norwalk-like viruses in hospitalized patients with gastroenteritis: evidence of nosocomial infections. J Clin Virol. 2000;17:151–8.
