# Supplementary material for: Systematic Literature Review of Role of Noroviruses in Sporadic Gastroenteritis
Source: Emerg Infect Dis. 2008 Aug;14(8):1224–31. doi: 10.3201/eid1408.071114 (PMC2600393; doi:10.3201/eid1408.071114)
Supplement: Technical Appendix 2 — References Used in Review but Not Cited in Article [file 07-1114_Techapp2-s2.pdf]

Systematic Literature Review of Role of Human Noroviruses in Sporadic Gastroenteritis

Technical Appendix 2

References Used in Review but Not Cited in Article

1. The World Bank. Country classification [cited 2008 Mar 3]. Available from http://go.worldbank.org/K2CKM78CC0

2. Parashar UD, Gibson CJ, Bresse JS, Glass RI. Rotavirus and severe childhood diarrhea. Emerg Infect Dis. 2006;12:304–6.

3. Greenberg HB, Valdesuso J, Kapikian AZ, Chanock RM, Wyatt RG, Szmuness W, et al. Prevalence of antibody to the Norwalk virus in various countries. Infect Immun. 1979;26:270–3.

4. Rockx B, De Wit M, Vennema H, Vinjé J, De Bruin E, Van Duynhoven Y, et al. Natural history of human calicivirus infection: a prospective cohort study. Clin Infect Dis. 2002;35:246–53.

5. Sakai Y, Nakata S, Honma S, Tatsumi M, Numata-Kinoshita K, Chiba S. Clinical severity of Norwalk virus and Sapporo virus gastroenteritis in children in Hokkaido, Japan. Pediatr Infect Dis J. 2001;20:849–53.

6. Iturriza Gomara M, Simpson R, Perault AM, Redpath C, Lorgelly P, Joshi D, et al. Structured surveillance of infantile gastroenteritis in East Anglia, UK: incidence of infection with common viral gastroenteric pathogens. Epidemiol Infect. 2008;136:23–33.

7. Trujillo AA, McCaustland KA, Zheng DP, Hadley LA, Vaughn G, Adams SM, et al. Use of TaqMan real-time reverse transcription-PCR for rapid detection, quantification, and typing of norovirus. J Clin Microbiol. 2006;44:1405–12.

8. Vinje J, Vennema H, Maunula L, von Bonsdorff CH, Hoehne M, Schreier E, et al. International collaborative study to compare reverse transcriptase PCR assays for detection and genotyping of noroviruses. J Clin Microbiol. 2003;41:1423–33.

9. Bailey MS, Boos CJ, Vautier G, Green AD, Appleton H, Gallimore CI, et al. Gastroenteritis outbreak in British troops, Iraq. Emerg Infect Dis. 2005;11:1625–8.

10. Tacket CO, Sztein MB, Losonsky GA, Wasserman SS, Estes MK. Humoral, mucosal, and cellular immune responses to oral Norwalk virus-like particles in volunteers. Clin Immunol. 2003;108:241–7.
